# Gastric inflammatory myofibroblastic tumor treated with combined laparoscopic and endoscopic gastric wedge resection: a case report

**DOI:** 10.1186/s12957-018-1460-0

**Published:** 2018-08-08

**Authors:** Masato Hayashi, Hirofumi Kawakubo, Shuhei Mayanagi, Rieko Nakamura, Koichi Suda, Norihito Wada, Yuko Kitagawa

**Affiliations:** Department of Surgery, School of Medicine, Keio University, 35 Shinanomachi, Shinjuku-ku, Tokyo, 160-8582 Japan

**Keywords:** Inflammatory myofibroblastic tumor, Stomach, Combined laparoscopic and endoscopic gastric wedge resection

## Abstract

**Background:**

Inflammatory myofibroblastic tumor is an uncommon soft tissue neoplasm rarely reported in the stomach.

**Case presentation:**

We identified a tumor highly suggestive of poorly differentiated gastric adenocarcinoma in the lesser curvature of the stomach of a 53-year-old female during screening endoscopy. Although the patient’s gastric biopsy did not reveal cancer, the tumor configuration was strongly suspicious for malignancy, and we performed a gastric wedge resection using a combined laparoscopic and endoscopic method. The lesion was diagnosed as inflammatory myofibroblastic tumor based on its morphological and immunohistological features.

**Conclusions:**

Inflammatory myofibroblastic tumor should be considered in the differential diagnosis of soft tissue tumors in the stomach. We present a case of inflammatory myofibroblastic tumor safely treated with combined laparoscopic and endoscopic gastric wedge resection.

## Background

Inflammatory myofibroblastic tumor (IMT) is an uncommon soft tissue neoplasm. The first reported IMT was identified in the lungs in 1937 [1]. IMT most commonly occurs in the pulmonary system of children and young adults [2] and exhibits variable biological behavior ranging from the typical benign type to more aggressive variants [3]. Gastric IMT is extremely rare, and there are no guidelines for its treatment. We present the case of a 53-year-old female with gastric IMT that was difficult to diagnose but effectively treated with combined laparoscopic and endoscopic gastric wedge resection.

## Case presentation

**Fig. 1:**
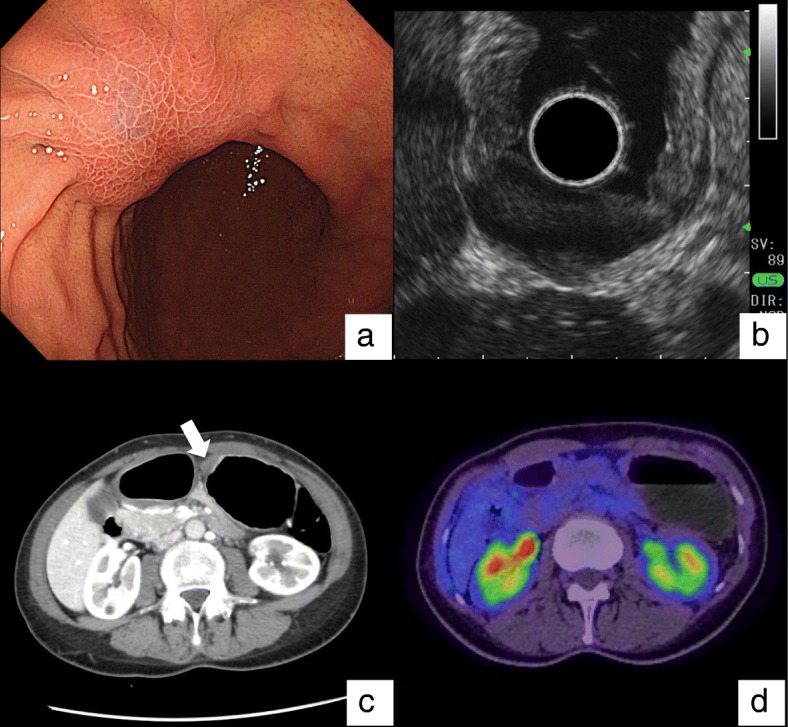
**a** Endoscopy reveals a flat protrusion in the lesser curvature of lower third of the stomach. **b** Endoscopic ultrasound shows the tumor thickening the second and third layers of the stomach wall. **c** Computed tomography (CT) scanning shows the thickened wall at the angle of the stomach (white arrow), but no evidence of tumor metastasis. **d** Positron emission tomography-CT shows no evidence of tumor metastasis or abnormal uptake in the tumor

A 53-year-old female presented with an asymptomatic gastric tumor found incidentally during screening upper gastrointestinal endoscopy. The lesion appeared as a flat protrusion in the lesser curvature of the lower third of the stomach (Fig. 1a). Endoscopic ultrasound (EUS) showed a 23 × 12-mm, hypovascular, heterogeneous lesion thickening the second and third gastric layers. The findings strongly suggested scirrhous gastric cancer (Fig. 1b). Although the lesion biopsy revealed no evidence of malignancy, our suspicion of scirrhous gastric carcinoma persisted due to the configuration of the tumor and our EUS findings. We performed an endoscopic open biopsy to make an accurate diagnosis. However, evaluation of the specimen revealed only slight chronic inflammatory cell invasion. Computed tomography (CT) scanning showed only gastric wall thickening at the angle of the stomach (Fig. 1c). Positron emission tomography-CT showed no evidence of metastasis or abnormal uptake in the tumor (Fig. 1d). The laboratory findings were normal as were levels of tumor markers (CEA, 1.6 ng/ml; CA19-9, 12 U/ml).

In spite of these findings, given our high level of suspicion, we decided to perform surgery to obtain a definitive diagnosis and treat the tumor. We chose gastric wedge resection using a combined laparoscopic and endoscopic method for several reasons. Firstly, the biopsy did not show cancer, but the tumor configuration strongly suggested malignancy; therefore, we selected a non-exposed method to prevent interoperative dissemination of tumor cells. Secondly, the tumor appeared to be a submucosal tumor (SMT), and gastric wedge resection using a combined laparoscopic and endoscopic method is among the safest procedures for resection of gastric SMTs [4, 5]. Lastly, we chose a wedge resection because if the tumor was not malignant, a distal gastrectomy could be considered excessive. We obtained an interoperative pathological diagnosis, and we planned to perform partial gastric resection in the absence of malignancy and laparoscopic distal gastrectomy with lymph node dissection if cancer was identified.

### Surgical procedure

The first port was inserted through the umbilicus using an open technique. Four additional ports were inserted: the second in the subcoastal arch, the third at the mid-point between the camera port and the second port, and the fourth port and fifth port symmetrically. The first, second, and third ports were 12 mm. The fourth and fifth ports were 5 mm.

**Fig. 2:**
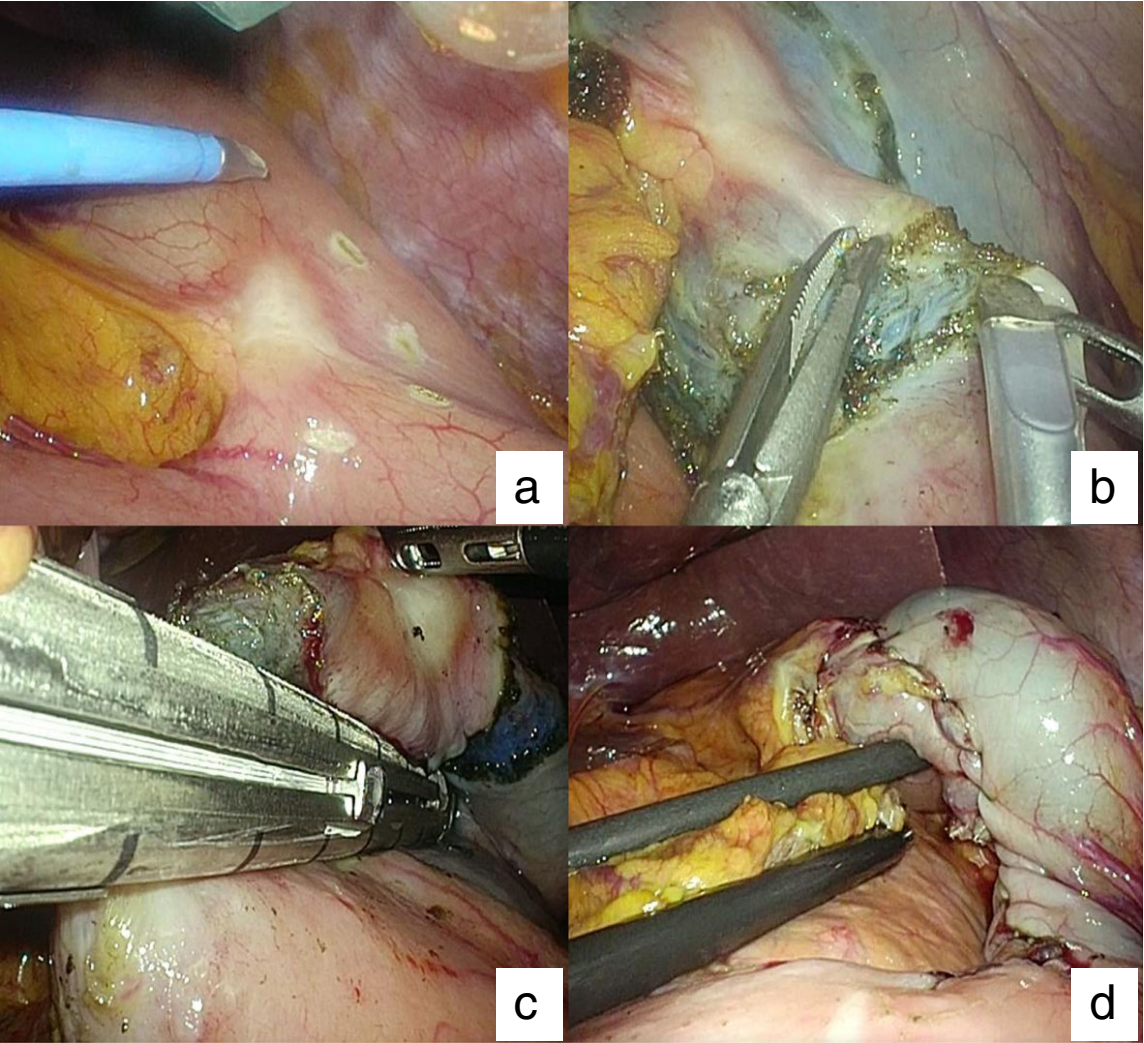
**a** The tumor circumference is marked to ensure an approximately 0.5-cm margin from the tumor edge using electrocautery. **b** Interoperative image showing the stomach after the seromuscular dissection was performed using a laparoscopic electrocautery scalpel. **c** The specimen is pulled up before performing full-thickness resection with a laparoscopic stapling device. **d** The seromuscular defect is sutured using a hand-sewn technique to avoid exposure of the staple line

The lesser curvature gastric tumor was easily recognized laparoscopically. Our intraabdominal examination revealed a scar-like tumor with twitch. After preparation of lesser curvature vessels, the tumor periphery was viewed endoscopically. The entire circumference of the tumor was marked to ensure an approximately 0.5-cm margin from the tumor edge (Fig. 2a). We then injected indigo carmine into the gastric submucosal layer with an endoscopic needle. The seromuscular dissection was performed using a laparoscopic electrocautery scalpel (Fig. 2b). The specimen was pulled up along with the surrounding mucosa. A full-layer resection including the specimen was achieved using a laparoscopic stapling device (Fig. 2c). We used a hand-sewn technique for seromuscular suturing so the staple line would not be exposed (Fig. 2d). Finally, we inserted the endoscope into the duodenum to ensure that there was no gastric stenosis.

### Clinical outcome

The patient had gastric hypoperistalsis on postoperative day (POD) 1. We inserted a gastric tube; however, we planned for an extended period with no oral intake in case of persistent hypoperistalsis. On POD 4, a W-elemental diet (W-ED) tube was inserted to drain the stomach and provide nutrition. We started the patient on continuous low-dose erythromycin, mosapride citrate, and Rikkunshi-Tou to treat her hypoperistalsis. The W-ED tube was removed on POD 11 after an oral contrast study confirmed gastric motility. On the same day, oral intake was initiated. The patient was discharged from the hospital on POD 18.

### Pathological findings

#### Interoperative pathological findings

The submucosa tumor could be seen in the resected specimen. The tumor had 20 mm × 20 mm × 13 mm size, including spindle cells with myxoid changes and collagen fibers. There was no evidence of adenocarcinoma. In this findings, fibromatosis can be a differential diagnosis.

#### Final pathological findings

**Fig. 3:**
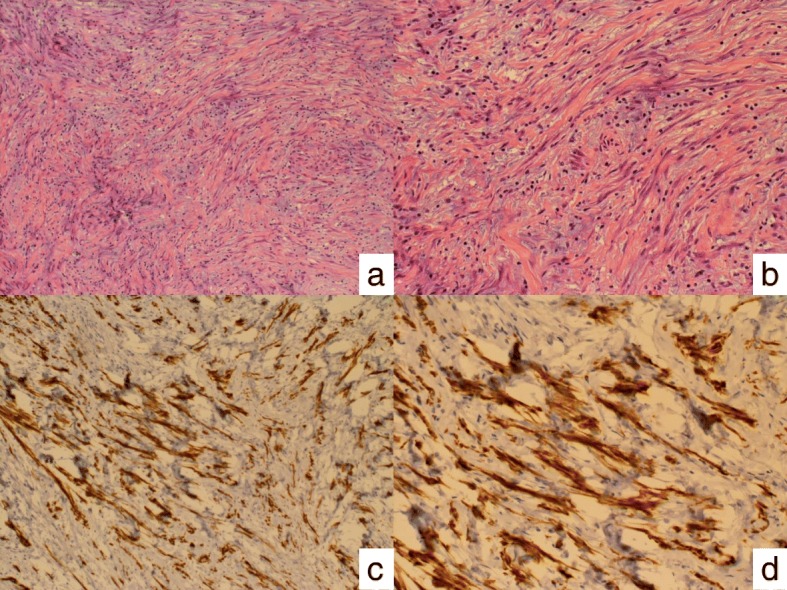
**a**, **b** Spindle tumor cells are present in the gastric wall from the submucosa to the serosa accompanied by myxoid changes and collagen fibers in the stroma (H&E; × 10, × 100). **c**, **d** The tumor cells are strongly immunopositive for anaplastic lymphoma kinase (× 10, × 100)

Spindle tumor cells were identified in the gastric wall from the submucosa to the serosa accompanied by myxoid changes and collagen fibers in the stroma (Fig. 3a and b). The spindle cells were strongly immunopositive for alpha-smooth muscle actin, and anaplastic lymphoma kinase (ALK), but immunonegative for c-kit, desmin, and S-100. CD34, bcl-2, beta-catenin, CD31, pankeratin, platelet-derived growth factor-A, and DOG1 were almost immunonegative (Fig. 3c and d). Based on these findings, the tumor was diagnosed as gastric IMT.

## Discussion and conclusions

IMT is considered an inflammatory pseudotumor, and there has been a debate as to whether it is benign or malignant [1, 3]. It is currently classified as an intermediate neoplasm according to the World Health Organization Histological Typing of Soft Tissue Tumors [6, 7]. IMT, although originally reported in the lung, is now recognized to occur in a variety of extrapulmonary sites ranging from the brain to the bladder [8]. Nevertheless, primary gastric IMT in adult is extremely rare [9], and almost all reported cases have been associated with symptoms such as abdominal pain or upper gastrointestinal bleeding [10]. Our case has two notable features: the method of treatment was gastric wedge resection using a combined laparoscopic and endoscopic method, and this patient had no symptoms.

Our review of 23 gastric IMT cases [9, 11–23] reported since 2000 is detailed in Table 1. The mean tumor size in the 23 cases was 7.4 cm. The most common surgical treatment was partial gastrectomy (9 cases, 39.1%) followed by partial gastrectomy combined with another organ resection (4 cases, 17.4%). Simultaneous resection was necessary in over 17% of cases because the IMT was found after it grew large enough to invade other organs. Only one case was treated by endoscopic mucosal resection. To the best of our knowledge, this report is the first to describe IMT treated with combined laparoscopic and endoscopic gastric wedge resection.

**Table 1:** Clinicopathologic features and surgery method in 23 cases of inflammatory myofibroblastic tumor

Age	
Range	2–80
Mean	35.9
Sex	
Male	11 (47.8%)
Female	12 (52.2%)
Tumor location	
Upper third	10 (43.5%)
Middle third	6 (26.1%)
Lower third	5 (21.7%)
Unknown	2 (8.7%)
Tumor size (cm)	
Range	1.8–15
Mean	7.4
Mitosis (HPF)	
Range	1–2
Mean	1
ALK	
Positive	8 (34.8%)
Negative	6 (26.1%)
Not available	9 (39.1%)
Surgery	
Distal gastrectomy	3 (13.0%)
Partial gastrectomy	9 (39.1%)
Total gastrectomy	1 (4.3%)
Wedge resection	1 (4.3%)
Partial gastrectomy with other organ resection	4 (17.4%)
Sleeve gastrectomy	1 (4.3%)
Combined laparoscopic and endoscopic gastric wedge resection	1 (4.3%)
EMR	1 (4.3%)
Unknown and other	2 (8.7%)

In this case, IMT was incidentally found on screening endoscopy. Fortunately, the lesion was relatively small and amenable to combined laparoscopic and endoscopic gastric wedge resection. The surgery was conducted safely with minimal blood loss and took 102 min. The tumor was completely resected from the patient’s stomach.

The IMT was ALK-positive. ALK expression may be associated with a more favorable prognosis in IMT [24–26], although consensus on this point has not yet been achieved. Because this tumor showed ALK expression and may have been found in an early stage, the patient’s prognosis might be favorable. Nine months after the surgery, this patient has no recurrence of the tumor. However, as IMT has malignant potential, the patient should continue follow-up CT and endoscopy.

We present a case of IMT that was very difficult to diagnose preoperatively. This lesion was safely and successfully treated with combined laparoscopic and endoscopic gastric wedge resection, a novel approach to IMT treatment. IMT should be considered in the differential diagnosis of a soft tissue gastric tumor, and combined laparoscopic and endoscopic gastric wedge resection could be a surgical treatment in cases of IMT.

## Abbreviations

ALK: Anaplastic lymphoma kinase
CT: Computed tomography
EMR: Endoscopic mucosal resection
EUS: Endoscopic ultrasound
HPF: High power field
IMT: Inflammatory myofibroblastic tumor
SMT: Submucosal tumor
W-ED: W-elemental diet
